# Is Altered Surfactant Protein Gene Expression in Peripheral Blood Associated with COVID-19 Disease Severity?

**DOI:** 10.3390/diagnostics15131690

**Published:** 2025-07-02

**Authors:** Suna Koc, Kamil Cankut Senturk, Sefa Cetinkaya, Guven Yenmis, Hulya Arkan, Mahmut Demirbilek, Pinar Acar, Erhan Arikan, Mehmet Dokur

**Affiliations:** 1 Department of Anesthesia and Reanimation, School of Medicine, Biruni University, Istanbul 34015, Turkey; 2 School of Medicine, Biruni University, Istanbul 34015, Turkey; 3 Department of Medical Genetics, School of Medicine, Biruni University, Istanbul 34015, Turkey; 4 Department of Medical Biology, Tayfur Ata Sokmen School of Medicine, Hatay Mustafa Kemal University, Hatay 31060, Turkey; 5 Center for Nanotechnology and Biomaterials Applications and Research, Marmara University, Istanbul 34854, Turkey; 6 Department of Emergency Medicine, School of Medicine, Biruni University, Istanbul 34015, Turkey; 7 Department of Emergency Medicine, Faculty of Medicine, Bilecik Seyh Edebali University, Bilecik 11230, Turkey

**Keywords:** COVID-19, surfactant protein, disease severity, gene expression, SFTPD

## Abstract

**Background/Objectives:** Severe COVID-19 pneumonia damages alveolar type II cells and disrupts surfactant homeostasis, contributing to acute respiratory distress syndrome (ARDS). Surfactant proteins (SP-A, SP-B, SP-C, SP-D) are critical for reducing alveolar surface tension and for innate immune defense. We aimed to evaluate whether surfactant protein gene expression varies with the severity of COVID-19. **Methods:** Peripheral blood was collected from 122 adults with confirmed COVID-19, categorized as asymptomatic (no symptoms), mild (requiring hospitalization), or severe (requiring ICU admission). We quantified mRNA expression of surfactant protein genes (SFTPA1, SFTPA2, SFTPB, SFTPC, SFTPD) in blood cells using RT-qPCR. Relative expression was normalized to GAPDH and compared among the groups using the 2^−ΔΔCt^ method. Outliers (Ct values > 3 SD from the mean) were excluded before analysis. **Results:** Distinct surfactant gene expression patterns were markedly associated with disease severity. Transcripts of SFTPB and SFTPC decreased with increasing severity of the disease. Notably, SFTPC expression was ~49-fold higher in mild cases compared to asymptomatic COVID-19-positive patients (*p* < 0.0001), but then decreased by ~54-fold in severe cases relative to mild (*p* < 0.0001), returning to near-baseline levels. In contrast, SFTPA2 and SFTPD were dramatically upregulated in severe cases. SFTPA2 was ~50-fold higher in severe versus mild cases (*p* < 0.0001), and SFTPD was ~4346-fold higher in severe versus asymptomatic cases (*p* < 0.0001; ~9.6-fold higher than in mild). SFTPA1 showed only a modest ~1.4-fold decrease in severe cases (vs. mild). All noted differences remained statistically significant after outlier exclusion. **Conclusions:** COVID-19 severity is correlated with profound changes in surfactant gene expression in blood. Critically ill patients exhibit loss of key surfactant components (SP-B and SP-C transcripts) alongside an excessive SP-D response. These preliminary findings suggest an imbalance that may contribute to lung injury in severe disease. However, further validation is needed to establish surfactant proteins, such as SP-D, as biomarkers of COVID-19 severity.

## 1. Introduction

COVID-19, caused by severe acute respiratory syndrome coronavirus 2 (SARS-CoV-2), is a respiratory infection first identified in December 2019. Declared a global pandemic by the World Health Organization (WHO) on 11 March 2020, the COVID-19 outbreak spread rapidly worldwide shortly after its emergence. During the first year of the pandemic, it infected approximately 30 million people across 212 countries, and to date, about 778 million cases have been reported, with roughly 9% of those infected having lost their lives [1].

COVID-19 infection can be seen in a clinical spectrum ranging from asymptomatic and mild cases to life-threatening critical illness [2,3]. The risk of severe disease varies according to age, underlying chronic diseases, and vaccination status [4]. Pre-vaccination studies have shown that asymptomatic infections account for 20–40% of all SARS-CoV-2 infections [5]. As of the end of May 2020, 1.3 million cases had been reported to the United States Centers for Disease Control and Prevention (CDC), with 14% hospitalized, 2% admitted to the intensive care unit, and 5% resulting in death [6]. Most people infected with SARS-CoV-2 experience mild to moderate illness. Severe and critical illness is marked by pneumonia that affects more than half of the lung tissue, rapid breathing, difficulty breathing, and oxygen levels below 94% in normal air.

The clinical course of COVID-19 ranges from mild symptoms to critical pneumonia. In particular, in high-risk groups, the infection can lead to severe inflammation in the lungs, increased capillary permeability, and consequent edema and microthrombi, eventually progressing to acute respiratory distress syndrome (ARDS) [7]. In most patients with COVID-19 pneumonia, lung pathology is compatible with acute respiratory distress syndrome (ARDS) [8]. In most autopsy reports, hyaline membrane changes and microvascular thrombosis, which are suggestive of early ARDS, have been reported [9]. Acute hypoxemic respiratory failure is the predominant finding in patients followed up in the intensive care unit with a diagnosis of ARDS [10]. The need for mechanical ventilation among patients in the intensive care unit varies between 20% and 100% [11].

During the early stages of the pandemic, the high transmissibility and mortality rates were attributed to the lack of knowledge about SARS-CoV-2 and the initially limited availability of specific treatments. Therefore, over the past few years, intensive research has been undertaken to clarify the pathogenesis of COVID-19 and to develop effective treatment strategies.

SARS-CoV-2 primarily uses the angiotensin-converting enzyme 2 (ACE2) receptor to enter host cells. The virus attaches to epithelial cells in the respiratory tract that express ACE2 and crosses the host cell membrane via fusion to initiate infection [12]. Alveolar epithelial cells responsible for gas exchange (alveolar type I, AT1) and those producing surfactants (alveolar type II, AT2) also express ACE2 on their surface. Indeed, one of the primary targets of the virus is AT2 cells, which become infected and damaged, leading to the disruption of alveolar integrity [13,14]. Damage to these cells results in decreased pulmonary surfactant production—a factor vital for alveolar function—causing alveolar collapse (atelectasis), ventilation–perfusion mismatch, and severe oxygenation disturbances that exacerbate ARDS [15]. Autopsy studies of COVID-19 patients have revealed severe damage to the alveolar–capillary barrier, hyaline membrane formation, widespread inflammation, microthrombi, and loss of surfactant proteins [16]. In other words, by targeting the alveoli, SARS-CoV-2 severely damages the pulmonary surfactant layer [17].

Pulmonary surfactant is a lipoprotein mixture that coats the inner surface of the alveoli, regulating the surface tension necessary for breathing and serving as the first line of defense against inhaled pathogens. It is composed of approximately 90% phospholipids (primarily dipalmitoylphosphatidylcholine) and about 10% proteins [18,19]. The primary protein components of surfactant are surfactant protein A (SP-A), surfactant protein B (SP-B), surfactant protein C (SP-C), and surfactant protein D (SP-D). SP-B and SP-C are small, hydrophobic, and amphipathic proteins that facilitate the formation of the alveolar surface phospholipid film, thereby reducing surface tension. In contrast, SP-A and SP-D are larger, hydrophilic collectin proteins responsible for the immune functions of surfactant [20].

Under normal conditions, SP-A and SP-D are present in the alveolar fluid and mucus layer, where they recognize inhaled pathogens. By binding to specific carbohydrate structures on the surface of these pathogens, they cause agglutination, thereby facilitating phagocytosis. These collectins also activate immune cells—especially alveolar macrophages—to clear pathogens and modulate the intensity of the inflammatory response when needed [21,22]. For example, SP-A has been shown to inhibit dendritic cell maturation and the release of IL-8 from eosinophils [23]. Thus, in addition to maintaining respiratory function, surfactant serves as an essential component of innate immunity.

Viral pneumonias and other lung infections can adversely affect surfactant synthesis and structure; indeed, during SARS-CoV-2 infection, significant disruptions occur in the pulmonary surfactant. With alveolar epithelial damage, surfactant production decreases, and the existing surfactant structure is also compromised in the inflammatory environment. In lung tissues of patients who have died from COVID-19, the expression of SP-A, SP-B, SP-C, and SP-D genes was reported to be markedly reduced, and this reduction was associated with high viral load. This is a consequence of the virus destroying the surfactant-producing AT2 cells. In moderate-to-severe COVID-19 cases, AT2 cell hyperplasia can be observed in the alveolar epithelium, and initial increases in SP-A and SP-D levels may occur. However, as the disease worsens, these protein levels decline again due to the exhaustion of surfactant synthesis [24].

Indeed, in COVID-19 patients, SP-D levels have been found to be significantly higher than those in control groups, particularly in cases of severe lung involvement, and to correlate with disease severity [25]. Similarly, plasma SP-A levels also increase in severe COVID-19 cases and have been proposed as a biomarker of lung injury [26]. These observations underscore the close relationship between surfactant protein levels and the severity of COVID-19.

On the other hand, the changes in hydrophobic surfactant proteins such as SP-B and SP-C during COVID-19 have not been fully elucidated. However, a recent study suggested that SP-B is associated with post-COVID lung damage and respiratory failure [27]. Immunohistochemical staining of lung samples from COVID-19 patients has revealed dense accumulations of SP-A in the alveolar spaces; these abnormal accumulations are believed to hinder the distribution of exogenously administered surfactant preparations, thereby reducing treatment efficacy [28].

Previous studies of COVID-19 have primarily focused on viral load and cytokine responses. The surfactant system, yet, is under-studied despite its importance in lung function. We therefore investigated whether surfactant protein gene expression is altered in COVID-19 patients with different severity levels. Ideally, one would sample lung tissues or perform bronchoalveolar lavage to assess alveolar surfactant expression; however, during the pandemic, such invasive procedures were impractical for most patients. Instead, we measured surfactant gene transcripts in peripheral blood as a proxy for lung involvement. Although pulmonary epithelial cells are the primary source of surfactant, transcripts or fragments may appear in blood through inflammatory processes (e.g., epithelial injury or exosomal transfer). Indeed, SFTPD mRNA has been detected in leukocytes in inflammatory conditions [29]. In this study, we compared the expression of SFTPA1, SFTPA2, SFTPB, SFTPC, and SFTPD in blood among asymptomatic, mild, and severe COVID-19 patients to identify patterns related to disease severity.

## 2. Materials and Methods

### 2.1. Sampling

A total of 162 adult patients (20–65 years) with laboratory-confirmed SARS-CoV-2 infection at Biruni University Hospital between March and November 2021 were enrolled. Confirmation of COVID-19 was performed by reverse transcription–quantitative PCR (RT-qPCR) by WHO diagnostic protocols. Ethical approval for this non-interventional study was granted by the Biruni University Non-Interventional Ethics Committee (Approval No. 2021/49-05), and all participants provided written informed consent prior to sample collection.

Participants were stratified into three clinical groups based on their symptoms and the level of care required. The asymptomatic COVID-19-positive patient group (*n* = 44; mean age, 30.3 ± 1.7 years) comprised outpatients with positive RT-qPCR results but no COVID-19-related symptoms and no requirement for hospitalization. The mild group (*n* = 48; mean age 33.8 ± 2.3 years) included symptomatic patients who required hospital admission but did not meet criteria for intensive care; and the severe group (*n* = 30; mean age 59.2 ± 2.8 years) included patients who required intensive care unit admission, mechanical ventilation, or who succumbed to the disease.

Severity grading followed WHO guidelines and was validated by a systematic review of each patient’s medical record. The overall cohort exhibited a balanced gender distribution (45% male) with no significant sex differences across groups. We excluded individuals younger than 20 or older than 65 years, pregnant or lactating women, patients with known immunosuppression (for example, chemotherapy recipients), and anyone unable to complete follow-up. Key comorbidities—including coronary artery disease, diabetes mellitus, hypertension, and smoking status—were recorded; the full demographic and clinical profile is summarized in Table 1. All enrolled participants had received the Pfizer-BioNTech COVID-19 vaccine and were monitored for 12 months post-diagnosis to assess disease progression and clinical outcomes.

### 2.2. Expression Analysis

Samples of 2 mL of peripheral venous blood were collected from each participant in EDTA tubes, and we immediately began total RNA isolation using the RNeasy Mini Kit (Qiagen, Cat. No. 74 106, Hilden, Germany) in strict accordance with the manufacturer’s instructions. Purified RNA was quantified, assessed for purity, and stored at −20 °C until further use. RNA quality was assessed by spectrophotometry (A260/A280 ratio) (NanoPhotometer P300, Implen GmbH, Munich, Germany), and only high-quality samples were included in the study.

For gene-expression analyses, 0.5 µg of each RNA sample was reverse-transcribed into cDNA using the QuantiTect Reverse Transcription Kit (Qiagen, Cat. No. 205 311, Hilden, Germany) following the manufacturer’s protocol. The transcript levels of SFTPA1 (OMIM#178630), SFTPA2 (OMIM#178642), SFTPB (OMIM#178640), SFTPC (OMIM#178620), and SFTPD (OMIM#178635) were then measured by real-time quantitative PCR with the QuantiTect SYBR Green PCR Kit (Qiagen, Cat. No. 154 027 284, Hilden, Germany). Gliseraldehit-3-Fosfat Dehidrogenaz gene (GAPDH) (OMIM#13400) was included as the internal housekeeping control. All qPCR assays were performed in technical triplicate, and any reactions exhibiting high variability among replicates were repeated to ensure data reliability.

All primer pairs were designed in silico using Primer3Plus to span exon–exon junctions and to minimize off-target amplification. The sequences for SFTPA1, SFTPA2, SFTPB, SFTPC, SFTPD, and GAPDH are detailed in Table 2. Amplification was performed under standard cycling conditions, and each reaction was performed in triplicate. Relative expression levels were calculated using the 2^−ΔΔCt^ method, normalizing each surfactant gene to GAPDH and then to the asymptomatic COVID-19-positive patient group.

### 2.3. Statistical Analysis

The statistical analyses were conducted using GraphPad Prism Software, versions 5.0 and 10.3 (GraphPad Software, San Diego, CA, USA). Age and gene expression levels are presented as mean ± standard error of the mean, as appropriate; categorical data are presented as counts and percentages. Group comparisons for age were performed using the Kruskal–Wallis test, whereas the Chi-square test or Fisher’s test was used for categorical variables. The Mann–Whitney U test was applied to compare gene expression levels between groups, and Spearman’s correlation was used to explore associations among gene expressions. A two-sided *p*-value of <0.05 was considered indicative of statistical significance.

Outliers (values beyond the mean ± 3 standard deviations) were excluded, and if a distribution was skewed only on one tail, the highest 5% of values were trimmed. These steps follow established outlier protocols. Effect sizes (r) were calculated from the Mann–Whitney Z-scores (r = Z/√N) to quantify the magnitude of group differences [30,31]. A post hoc power analysis was also conducted using G*Power 3.1.9.7 (Universität Düsseldorf) for each comparison, based on the observed effect sizes.

## 3. Results

In our study, 44 asymptomatic COVID-19-positive patients (with no symptoms), 48 symptomatic patients (with mild symptoms but no need for intensive care unit), and 30 severe cases (requiring intensive care unit) were included. All individuals were COVID-19-positive. The demographic data, comorbidities, and symptomatic characteristics of the cases are presented in Table 3. (Note: asymptomatic individuals were COVID-19-positive, so they serve as a reference but are not accurate healthy controls).

When evaluating the age differences among groups, it was observed that as the severity of the disease increased, there was a statistically significant increase in the mean age across all group comparisons (*p* < 0.0001).

Among the asymptomatic COVID-19-positive patient group, 40.9% were male and 59.1% were female; in the mild group, 37.5% were male and 62.5% were female; and in the severe group, 63.3% were male and 36.7% were female. No statistically significant difference was found in gender distribution among the groups (*p* = 0.0652).

Overall, 43.2% of the asymptomatic COVID-19-positive patient group, 29.2% of the mild group, and 83.3% of the severe group were smokers. A significant difference in smoking rates was found among the groups (*p* < 0.0001).

A statistical comparison revealed a significant difference in HT among the groups (*p* = 0.0009); however, no significant differences were observed for DM and CAD (*p* = 0.4797 and *p* = 0.0833, respectively). Significant differences were found among the three groups regarding fever, fatigue, sepsis, and ARDS (*p* < 0.0001), while no significant difference was observed for BP (*p* = 0.2312) (Table 3).

**Figure 1:**
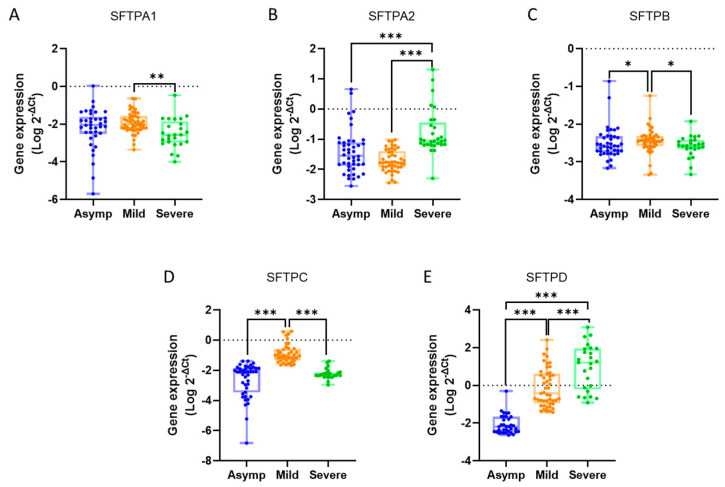
Relative gene expression levels of (**A**) SFTPA1, (**B**) SFTPA2, (**C**) SFTPB, (**D**) SFTPC, and (**E**) SFTPD across the three groups. Each dot represents the Log 2^−ΔCt^ value, and the bar indicates the range from minimum to maximum. The Mann–Whitney U test was used for pairwise comparisons between groups. * *p* ≤ 0.05, ** *p* ≤ 0.01, *** *p* ≤ 0.001.

The mean expression values for the studied genes across the groups were as follows: SFTPA1 showed levels of 0.0461 ± 0.0270 in the asymptomatic COVID-19-positive patient group, 0.0271 ± 0.0069 in the mild group, and 0.0199 ± 0.0136 in the severe group. For SFTPA2, the expression was 0.2635 ± 0.1272 in asymptomatic COVID-19-positive individuals, 0.0271 ± 0.0035 in the mild group, and 1.3530 ± 0.7601 in severe cases. SFTPB exhibited values of 0.0075 ± 0.0034, 0.0052 ± 0.0012, and 0.0030 ± 0.0004 in the asymptomatic COVID-19-positive, mild, and severe groups, respectively. The expression of SFTPC was 0.0092 ± 0.0016 for asymptomatic COVID-19-positive individuals, 0.4503 ± 0.1464 for the mild group, and 0.0084 ± 0.0019 for severe cases. Lastly, SFTPD demonstrated levels of 0.0252 ± 0.0140 in the asymptomatic COVID-19-positive patient group, 11.430 ± 5.8230 in the mild group, and 109.30 ± 52.20 in the severe group (Figure 1).

When comparing gene expression levels between groups, SFTPA1 showed a 1.36-fold very significant decrease in the severe group relative to the mild group (*p* = 0.0013). For SFTPA2, expression increased 5.13-fold in the severe group compared to the asymptomatic COVID-19-positive patient group and 50.02-fold compared to the mild group, both changes being highly significant (*p* < 0.0001).

The expression of SFTPB decreased significantly by 1.44-fold in the mild group compared to the asymptomatic COVID-19-positive patient group (*p* = 0.0482) and by 1.73-fold in the severe group compared to the mild group (*p* = 0.0120). For SFTPC, there was a 48.74-fold highly significant increase in the mild group compared to the asymptomatic COVID-19-positive patient group (*p* < 0.0001), followed by a 53.84-fold highly substantial decrease in the severe group relative to the mild group (*p* < 0.0001).

Finally, SFTPD expression increased by 454.47-fold in the mild group compared to the asymptomatic COVID-19-positive patient group, 4345.92-fold in the severe group compared to the asymptomatic COVID-19-positive patient group, and 9.56-fold in the severe group relative to the mild group (*p* < 0.0001, *p* < 0.0001, and *p* = 0.0002, respectively) (Table 4).

To assess the robustness of our findings given the sample sizes, we performed post hoc power analysis (Table 4). For each pairwise group comparison and each gene, we report the effect size (r) and statistical power (1-β). Several key comparisons had power ≥ 0.80. For example, SFTPC (severe vs. mild) had r = 0.82 (power ≈ 0.93), and SFTPD (asymptomatic vs. mild) had r = 0.84 (power ≈ 0.97). In contrast, comparisons such as SFTPA1 (severe vs. mild) yielded a correlation coefficient of r = 0.48 (power ≈ 0.51), indicating a limited ability to detect differences. These power estimates confirm that our strongest results (high fold changes in SP-C and SP-D) were supported by an adequate sample size, whereas subtler effects should be interpreted more tentatively. Overall, severe COVID-19 was associated with a loss of SP-B/SP-C transcripts and a substantial rise in SP-D (and SP-A2) transcripts.

A strong positive correlation was identified between the gene pairs SFTPA1-SFTPA2 and SFTPC-SFTPD in the mild group, with correlation coefficients of r = 0.673 (*p* < 0.0001) and r = 0.722 (*p* < 0.0001), respectively. This indicates that the expression levels of these genes tend to rise or fall together under similar conditions, suggesting potential co-regulation or shared functional roles (Table 5).

In contrast, medium negative correlations were observed between the pairs SFTPA1-SFTPC, SFTPA2-SFTPC, and SFTPA2-SFTPD in the mild group, with coefficients of r = −0.492 (*p* = 0.0015), r = −0.537 (*p* = 0.0004), and r = −0.499 (*p* = 0.0004), respectively. These inverse relationships suggest that these gene pairs have distinct regulatory or functional mechanisms (Table 5).

For SFTPA1 and SFTPD, the correlation shifted based on disease severity. A weak negative correlation was noted in the asymptomatic group (r = −0.396, *p* = 0.0273), progressing to a medium negative correlation in the mild group (r = −0.549, *p* = 0.0001), and culminating in a strong negative correlation in the intensive care group (r = −0.653, *p* = 0.0007). This pattern highlights an intensifying inverse relationship as disease severity increases, suggesting these genes may have distinct and potentially opposing roles in the pathophysiology of COVID-19. The findings point to a dynamic interplay between these genes that becomes more pronounced with worsening clinical conditions (Table 5).

## 4. Discussion

Mortality in a COVID-19 infection has been associated with the presence of severe ARDS and varies between 12% and 78%, with an average of 25–50% [32]. As the pandemic progressed and vaccination rates increased, mortality rates decreased [33]. The most critical risk factor associated with mortality in critically ill patients is advanced age. Severe ARDS development and the need for mechanical ventilation, comorbid chronic diseases (obesity, hypertension, chronic heart and lung diseases, cancer), right ventricular dysfunction, and male gender are risk factors associated with mortality [34]. In a study involving more than 2000 critically ill patients, an 11-fold increase in mortality rates over the age of 80 was reported [35]. Worse still, ARDS caused by COVID-19 with reduced pulmonary surfactant levels may severely impair alveolar collapse, gas exchange, and increase the work of breathing [36]. According to this, COVID-19 patients with severe pneumonia requiring intensive care follow-up in our study were predominantly male and relatively middle-aged, had a high number of cardiovascular comorbidities, and were mainly patients with signs of sepsis, which is consistent with the current literature on this subject.

In a large-scale study by Salvioni et al., plasma SP-D levels were identified as a biomarker for predicting COVID-19 diagnosis and mortality, with threshold values of 150 ng/mL and 250 ng/mL indicating the presence of disease and the risk of death, respectively [25]. Our data suggest that SP-D is markedly elevated in severe COVID-19, consistent with prior reports that surfactant protein D is a severity indicator [37]. However, we measured mRNA rather than protein, and we did not establish the predictive value of SP-D; thus, we consider it a preliminary signal rather than a validated biomarker.

Our findings on the SFTPA genes are parallel to the complex picture in the literature. In our study, SFTPA2 (SP-A2) expression was markedly increased in critically ill patients requiring intensive care, whereas SFTPA1 (SP-A1) expression showed a declining trend. Some studies have reported that SP-A protein levels rise as COVID-19 pneumonia progresses, and that SP-A could be a crucial marker in the early stages of lung injury. In one such study, the authors suggested that although SP-A and SP-D serum levels in COVID-19 patients were higher than in healthy individuals, these levels might drop again in very advanced disease due to alveolar type II cell destruction [38]. On the other hand, another study found no significant difference in SP-A levels between severe and mild cases [39]. Our results highlight a divergence in the behavior of SP-A genes. In other words, we observed an increase in SP-A2 expression, whereas a decrease in SP-A1. The reduction in SP-A1 and increase in SP-A2 might reflect the differentiated immune response described in the literature. Indeed, Floros and Phelps propose that SP-A1 and SP-A2 have different effects in COVID-19 and may influence the disease outcome in other various ways [40].

SFTPC (SP-C) gene expression exhibited a notable biphasic pattern in our study: in mild patients, it showed a dramatic rise, whereas in the severe group, it fell sharply to near the levels of asymptomatic COVID-19-positive patients. This change is in line with some observations, as moderate COVID-19 pneumonia has been associated with alveolar type II cell hyperplasia and increased surfactant production [28]. At that stage, the lung may be attempting to compensate for injury via increased surfactant synthesis; indeed, the 50-fold increase in SFTPC in our mild group indicates a functional alveolar response. However, as disease severity increases and diffuse alveolar damage with hyaline membrane formation develops, SFTPC expression falls markedly due to the loss of type II cells [41]. Similarly, a COVID-19 lung transcriptome analysis by Islam and Khan revealed that SARS-CoV-2 infection suppresses the expression of surfactant protein genes [42]. The drop in SFTPC observed in our severe group supports the notion that the virus severely impairs surfactant-producing cell function. Overall, these findings support the general idea that in severe cases, the surfactant system fails, leading to gas exchange impairments, resulting in impaired gas exchange. A study in pediatric patients similarly reported no significant correlation between plasma SP-B levels and COVID-19 severity [43]. This is likely due to the limited passage of SP-B into the bloodstream and the difficulty of detecting it. The changes we observed align with the known biology of surfactant proteins. SP-B and SP-C are hydrophobic proteins synthesized by AT2 cells, and they are critical for maintaining mechanical lung stability. Their near-loss of transcripts likely reflects extensive damage to AT2 cells in severe COVID-19 [13,14]. Indeed, decreased pulmonary surfactant is implicated in the pathogenesis of ARDS [15]. We also note that SP-B and SP-C proteins are challenging to detect in blood, even in the presence of lung injury [44], which is consistent with our finding of low and non-distinct SFTPB/SFTPC signals and limited power for those comparisons. Indeed, even in ARDS patients, measuring SP-B in serum requires exceptional sensitivity; SP-B is present in blood only at ng/mL levels and can only be measured with advanced methods [45]. Our qPCR-based approach to indirectly measure gene expression reflected this difficulty as well; SFTPB and SFTPC transcript levels were extremely low in all groups, and although their changes were statistically significant, the magnitude of change was limited.

The structural differences among surfactant proteins influence their presence in circulation: SP-A and SP-D are water-soluble collectins, whereas SP-B and SP-C are extremely hydrophobic [44]. In contrast, SP-A and SP-D are collectins with immunomodulatory functions. SP-D, in particular, binds pathogens and damaged cells to modulate inflammation. The SFTPD upregulation we found in severe patients is consistent with this role. It may seem counterintuitive that surfactant gene transcripts were detectable in blood. However, severe lung injury in COVID-19 (ARDS) can lead to alveolar barrier disruption, resulting in the release of cellular material into the circulation. Recent evidence shows that patients with ARDS have high levels of lung-specific mRNA in their blood, correlating with disease severity [46]. This suggests that damaged alveolar cells release mRNA (as cell-free RNA or within extracellular vesicles) into the bloodstream. Additionally, one of these prior studies has detected circulating pulmonary epithelial cells or their mRNAs in lung fibrosis patients [47], supporting the concept that lung-derived genetic material can be found in peripheral blood. Extracellular vesicles (exosomes) are a plausible carrier of such signals—it has been hypothesized that the lung “signals” systemic inflammation via vesicles containing lung-specific RNAs [46]. Together, these mechanisms could explain why we observe changes in surfactant gene expression in blood, reflecting pulmonary expression changes in COVID-19.

Consequently, SP-B and SP-C levels or expression did not show as pronounced changes as those of SP-A and SP-D. It appeared less useful for distinguishing COVID-19 severity. This aligns with the general view that in COVID-19, the most notable changes occur in the hydrophilic surfactant proteins (which have immunological functions), whereas changes in the hydrophobic proteins remain unclear due to detection challenges [47].

However, it is essential to emphasize that our measurements are at the mRNA level in blood, not direct protein quantification in lungs. However, the transcripts could originate from circulating immune cells, from epithelial cells released into circulation, or from extracellular vesicles. Additionally, supporting mRNA-level changes with existing literature on corresponding protein-level alterations is a commonly employed strategy. This approach allows researchers to reasonably argue that, even in the absence of direct protein validation, the observed transcriptomic shifts likely reflect underlying changes at the protein level under similar pathological conditions. Nonetheless, the direction of change aligns with what might be expected: a loss of surfactant-producing cells (lower SFTPB/SFTPC mRNA) and an activation of innate immunity (higher SFTPA2/SFTPD mRNA). This suggests that peripheral blood transcripts can serve as a partial mirror of pulmonary events. As supported by Wojcik et al. (2016) [29], elevated SFTPD mRNA was detected in blood leukocytes of patients with gestational diabetes. Further evidence, as demonstrated by Park et al. (2017), showed that surfactant protein genes can also be detected in circulation during inflammatory conditions [48]. Clarifying this will require future work (e.g., sorting cell types, proteomic assays).

## 5. Conclusions

This study aimed to elucidate the effect of COVID-19 on the lung’s surface tension regulatory system by examining the expression of surfactant protein genes (SFTPA1, SFTPA2, SFTPB, SFTPC, and SFTPD) across different disease severity groups. By focusing on a relatively new topic and directly evaluating surfactant gene expression in patient samples, the study holds a unique place in the literature, even though the presented data is preliminary. Traditionally, COVID-19 research has concentrated on the cytokine storm, immune response, or viral load; in contrast, this study demonstrates surfactant system disruption with quantitative gene expression data, filling a significant gap. It provides concrete evidence of disrupted surfactant homeostasis in COVID-19. The findings show that in severe cases, some surfactant genes are significantly suppressed, whereas others (especially SFTPA2 and SFTPD) are abnormally elevated. In particular, the exponential rise in SFTPD expression in critical patients suggests that this protein may be an indicator of tissue damage and inflammatory response.

However, this study has some limitations. First, the sample is from a single center and includes a limited number of patients with severe conditions. This study was conducted under significant logistical constraints imposed by the COVID-19 pandemic, which limited sample availability and recruitment. As such, the number of patients within each severity subgroup was relatively small, and we acknowledge that this may reduce the statistical power of our comparisons. Second, although measuring surfactant gene expression in peripheral blood is innovative, it is unclear to what extent these data accurately reflect actual surfactant production in lung tissue; the source of the transcripts detected in blood (circulating immune cells vs. material from damaged alveolar cells) has not been fully determined. Third, the cross-sectional design of the study does not permit assessment of dynamic changes in expression over time or determination of cause-and-effect relationships. While all patients were vaccinated—which gives this cohort a unique characteristic—it remains unknown whether similar results would apply to unvaccinated patients or those infected with different viral variants. Finally, this study lacks a truly healthy control group, as all participants were COVID-19 positive. Asymptomatic individuals were used as a reference baseline, but they may not reflect normal physiology. This limits our ability to discern disease-specific changes; therefore, our results should be interpreted with caution. The decision to forgo external controls was due to pandemic constraints. Still, future work must include healthy and non-COVID ARDS controls to validate the specificity of these surfactant gene changes.

In conclusion, we present an exploratory analysis of surfactant protein gene expression in COVID-19 blood samples, revealing significant alterations related to disease severity. These findings generate new hypotheses about surfactant dysfunction in systemic illness. While they support the concept of surfactant involvement in COVID-19 pathophysiology, they are not definitive. Further research with larger cohorts, appropriate controls, and direct lung tissue comparisons will be needed to confirm our observations and assess their clinical relevance.

## Figures and Tables

**Table 1:** The demographic distribution of the participants.

	Asymp (*n* = 44)	Mild (*n* = 48)	Severe (*n* = 30)
Age (years)	30.3 ± 1.7	33.8 ± 2.3	59.2 ± 2.8
Sex			
Male	18	18	19
Female	26	30	11
Smoking status	19	14	25
Comorbidities			
HT	7	6	14
DM	6	3	2
CAD		1	3
Symptoms			
Fever		41	3
Fatigue		42	2
Sepsis		1	27
ARDS		1	24
BP		2	2

Data were shown as mean ± standard error of the mean, or number. HT, Hypertension; DM, Diabetes Mellitus; CAD, Coronary Artery Disease; ARDS, Acute Respiratory Distress Syndrome; BP, bronchopneumonia.

**Table 2:** The primer sequences for the expression analysis.

Name	Sequence (5′–3′)
SFTPA1_sense	CTCCTGGAAATGATGGGCTGC
SFTPA1_antisense	GTCTAAAGTCGTGGAGTGTGGC
SFTPA2_sense	TGGAGAGCGTGGAGAGAAGG
SFTPA2_antisense	TGATGTCTGAAGTCGTGGAGTG
SFTPB_sense	CACCTCATCCTTGGCCTGTG
SFTPB_antisense	CTTGGCATAGGTCATCGGCTC
SFTPC_sense	GCCTTCTTATCGTGGTGGTGG
SFTPC_antisense	TGGTAACCAGGTGCTCACTCA
SFTPD_sense	GGAGCAAAGGGAGAAAGTGGG
SFTPD_antisense	CTGAGAGAAAGCAGCCTGGAG
GAPDH_sense	GAGTCAACGGATTTGGTCGT
GAPDH_antisense	GACAAGCTTCCCGTTCTCAG

**Table 3:** Demographic evaluation of the participants.

	Asymp (*n* = 44)	Mild (*n* = 48)	Severe (*n* = 30)	*p*-Value
Age (years)	30.3 ± 1.7	33.8 ± 2.3	59.2 ± 2.8	**<0.0001** ^a^
Sex				
Male	18 (40.9)	18 (37.5)	19 (63.3)	0.0652 ^b^
Female	26 (59.1)	30 (62.5)	11 (36.7)	
Smoking status (%)	19 (43.2)	14 (29.2)	25 (83.3)	**<0.0001** ^b^
Comorbidities				
HT	7 (15.9)	6 (12.5)	14 (46.7)	**0.0009** ^b^
DM	6 (13.6)	3 (6.3)	2 (6.7)	0.4797 ^c^
CAD	0 (0)	1 (2.1)	3 (10)	0.0833 ^c^
Symptoms				
Fever	0 (0)	41 (85.4)	3 (10)	**<0.0001** ^b^
Fatigue	0 (0)	42 (87.5)	2 (6.7)	**<0.0001** ^b^
Sepsis	0 (0)	1 (2.1)	27 (90)	**<0.0001** ^b^
ARDS	0 (0)	1 (2.1)	24 (80)	**<0.0001** ^b^
BP	0 (0)	2 (4.2)	2 (6.7)	0.2312 ^c^

Data are presented as mean ± standard error of the mean, or number (percent). HT, hypertension; DM, diabetes mellitus; CAD, coronary artery disease; ARDS, Acute respiratory distress syndrome; BP, Bronchopneumonia. Values with *p* < 0.05 are highlighted in bold. ^a^ Kruskal–Wallis Test, ^b^ Chi Square Test, ^c^ Fisher Exact Test.

**Table 4:** Relative comparison of gene expression among the groups.

	Mild/Asympt				Severe/Asympt				Severe/Mild			
Genes	Fold Change	*p*-Value	Effect Size	Power (1-β)	Fold Change	*p*-Value	Effect Size	Power (1-β)	Fold Change	*p*-Value	Effect Size	Power (1-β)
SFTPA1	−1.70	0.3997	0.21	0.16	−2.31	0.0632	0.39	0.36	−1.36	**0.0013**	0.48	0.51
SFTPA2	−9.74	0.0663	0.22	0.18	5.13	**<0.0001**	0.48	0.50	50.02	**<0.0001**	0.72	**0.85**
SFTPB	−1.44	**0.0482**	0.27	0.23	−2.50	0.7874	0.12	0.08	−1.73	**0.0120**	0.35	0.31
SFTPC	48.74	**<0.0001**	0.84	**0.97**	−1.10	0.9741	0.18	0.11	−53.84	**<0.0001**	0.82	**0.93**
SFTPD	454.47	**<0.0001**	0.84	**0.97**	4345.92	**<0.0001**	0.84	**0.93**	9.56	**0.0002**	0.52	0.58

The 2^−ΔCt^ values were compared among the groups. The Mann–Whitney U test was applied, and values with *p* < 0.05 are highlighted in bold. Results of effect size and power analysis for pairwise comparison. Comparisons in bold have power ≥ 0.80.

**Table 5:** Evaluation of the correlation between gene expression levels across all groups.

	Asymptomatic		Mild		Severe	
Gene Pair	r	*p*-Value	r	*p*-Value	r	*p*-Value
SFTPA1-SFTPA2	0.307	0.0541	0.673	**<0.0001**	0.020	0.9258
SFTPA1-SFTPB	−0.080	0.6345	0.102	0.5062	0.370	0.0748
SFTPA1-SFTPC	0.001	0.9946	−0.492	**0.0015**	−0.049	0.8281
SFTPA1-SFTPD	−0.396	**0.0273**	−0.549	**0.0001**	−0.653	**0.0007**
SFTPA2-SFTPB	0.024	0.8781	0.037	0.8105	−0.033	0.8671
SFTPA2-SFTPC	0.058	0.7115	−0.537	**0.0004**	−0.059	0.7785
SFTPA2-SFTPD	−0.092	0.5974	−0.499	**0.0004**	0.074	0.7243
SFTPB-SFTPC	−0.013	0.9341	−0.083	0.6126	0.050	0.8150
SFTPB-SFTPD	0.046	0.7949	−0.254	0.0927	−0.230	0.2787
SFTPC-SFTPD	0.161	0.3705	0.772	**<0.0001**	−0.037	0.8712

Spearman’s test was applied. r represents the correlation coefficient. Values with *p* < 0.05 are highlighted in bold.

## Data Availability

The original contributions presented in this study are included in the article. Further inquiries can be directed to the corresponding author.
